# Integrative study of pandemic A/H1N1 influenza infections: design and methods of the CoPanFlu-France cohort

**DOI:** 10.1186/1471-2458-12-417

**Published:** 2012-06-07

**Authors:** Nathanael Lapidus, Xavier de Lamballerie, Nicolas Salez, Michel Setbon, Pascal Ferrari, Rosemary M Delabre, Marie-Lise Gougeon, Frédéric Vely, Marianne Leruez-Ville, Laurent Andreoletti, Simon Cauchemez, Pierre-Yves Boëlle, Eric Vivier, Laurent Abel, Michaël Schwarzinger, Michèle Legeas, Pierre Le Cann, Antoine Flahault, Fabrice Carrat

**Affiliations:** 1 Institut National de la Santé et de la Recherche Médicale, UMR-S 707, F-75012 Paris, France; 2 Université Pierre et Marie Curie-Paris 6, UMR-S 707, F-75012 Paris, France; 3 Unité des Virus Emergents, UMR-D 190, Aix-Marseille université and Institut de Recherche pour le Développement, Marseille, France; 4 Laboratoire de Virologie, Pôle hospitalier de Microbiologie et Maladies Infectieuses, Assistance Publique, Hôpitaux de Marseille, Marseille, France; 5 Ecole des Hautes Etudes en Sante Publique, Rennes, France; 6 CNRS – LEST, UMR 6123 Université d’Aix-Marseille, Aix en Provence, France; 7 Ecole des Hautes Etudes en Sante Publique, Paris, France; 8 Institut Pasteur, Antiviral Immunity, Biotherapy and Vaccine Unit, Paris, France; 9 Centre d’Immunologie de Marseille-Luminy (CIML), Université de la Méditerranée UM 631, Campus de Luminy, 13288 Marseille, France; 10 Institut National de la Santé et de la Recherche Médicale, UMR-S 631, Marseille, France; 11 CNRS, UMR 6102, Marseille, France; 12 Assistance Publique, Hôpitaux de Marseille, Hôpital de la Conception, Marseille, France; 13 Université Paris Descartes, Sorbonne Paris Cité, EA 36-20 Paris, France; 14 Laboratoire de Virologie, Hôpital Necker, AP-HP, Paris, France; 15 Unité de Virologie Médicale et Moléculaire, Centre Hospitalier Universitaire, Reims, France; 16 IFR 53/EA-4303 (DAT/PPCIDH), Faculté de Médecine, Reims, France; 17 Medical Research Council Centre for Outbreak Analysis and Modeling, Department of Infectious Disease Epidemiology, Imperial College, London, UK; 18 Assistance Publique-Hôpitaux de Paris, Hôpital Saint Antoine, Unité de Santé Publique, F-75012 Paris, France; 19 Laboratoire de Génétique Humaine des Maladies Infectieuses, Institut National de la Santé et de la Recherche Médicale, U 550, Paris, France; 20 Laboratory of Human Genetics of Infectious Diseases, Rockefeller Branch, Rockefeller University, New York, NY, USA; 21 Institut National de la Santé et de la Recherche Médicale, U 912, Marseille, France; 22 Université Aix Marseille, IRD, UMR-S912, Marseille, France; 23 Observatoire Régional de la Santé PACA, Marseille, France

**Keywords:** Influenza a virus H1N1 subtype, Cohort study, Risk factors, France

## Abstract

**Background:**

The risk of influenza infection depends on biological characteristics, individual or collective behaviors and the environmental context. The Cohorts for Pandemic Influenza (CoPanFlu) France study was set up in 2009 after the identification of the novel swine-origin A/H1N1 pandemic influenza virus. This cohort of 601 households (1450 subjects) representative for the general population aims at using an integrative approach to study the risk and characteristics of influenza infection as a complex combination of data collected from questionnaires regarding sociodemographic, medical, behavioral characteristics of subjects and indoor environment, using biological samples or environmental databases.

**Methods/Design:**

Households were included between December 2009 and July 2010. The design of this study relies on systematic follow-up visits between influenza seasons and additional visits during influenza seasons, when an influenza-like illness is detected in a household via an active surveillance system. During systematic visits, a nurse collects individual and environmental data on questionnaires and obtains blood samples from all members of the household. When an influenza-like-illness is detected, a nurse visits the household three times during the 12 following days, and collects data on questionnaires regarding exposure and symptoms, and biological samples (including nasal swabs) from all subjects in the household. The end of the follow-up period is expected in fall 2012.

**Discussion:**

The large amount of data collected throughout the follow-up will permit a multidisciplinary study of influenza infections. Additional data is being collected and analyzed in this ongoing cohort. The longitudinal analysis of these households will permit integrative analyses of complex phenomena such as individual, collective and environmental risk factors of infection, routes of transmission, or determinants of the immune response to infection or vaccination.

## Background

The first human cases of influenza caused by a novel swine-origin A/H1N1 pandemic influenza virus variant (H1N1pdm) were reported in Mexico and the United States in April 2009 [1]. Given the rapid spread of this virus and considering the likelihood of its pandemic extent – confirmed by the World Health Organization (WHO) on June 11, 2009 [2] – the Cohorts for Pandemic Influenza (CoPanFlu) international consortium was initiated to study individual and collective determinants of H1N1pdm influenza infection across countries by setting up prospective cohorts of households, followed for 2 years in 6 countries or regions of the world: metropolitan France, Mali [3], Bolivia, Laos, Reunion Island [4] and Djibouti. The CoPanFlu-France cohort, set up in metropolitan general population, is part of the CoPanFlu international consortium and its protocol served as a blueprint for the other international cohorts.

Several studies already reported risk factors for seasonal or pandemic influenza infection in households. These studies focused on including individual characteristics of index patients and their household contacts [5-10] or hygiene measures as predictors of secondary household infections [11,12]. In addition to household studies, risk factors of seasonal influenza infections have been studied in relation to characteristics of social contacts [13] or baseline serological status of the host [14]. However, to our knowledge, no attempt was made to study the risk of influenza infection as a complex combination of biological characteristics (including immunity), individual or collective behaviors and environmental context. This integrative approach, in which epidemiological data is comprehensively collected and analyzed, is currently developed for non-communicable diseases and relies on methods derived from Genome-Wide Association Studies (GWAS) [15,16]. To achieve our objectives, we developed a multidisciplinary approach, with an original design involving data collection on subjects and their environment and biological samples.

## Methods/Design

### Sampling

This cohort was designed to assess the relative risk of infection by the H1N1pdm virus. We first intended to include 1000 households (about 2100 subjects) which would have permitted to detect covariates associated to a relative risk ≥ 1.4 with a 80% power and 5% significance, assuming a cumulative incidence of 10% and intra-household correlation of 0.3.

**Figure 1:**
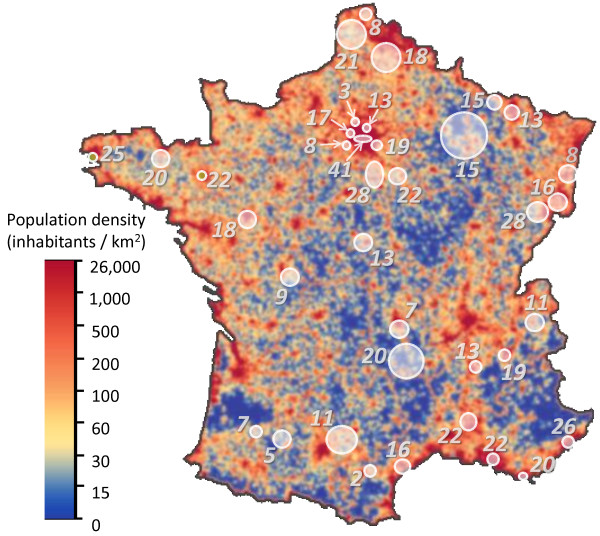
**Distribution of included households in relation to density of population (total: N = 601).** White discs represent the 40 areas of the study. Overlapping areas are merged.

Households were sampled using a random telephonic procedure (Mitofsky–Waksberg design [17]) in a stratified geographical sampling scheme, aimed at including a sample of subjects as close as possible to the French general population [18,19]. Forty addresses were drawn from the national directory. These addresses defined the centers of 40 areas inside which subjects were eligible. The limits of these areas were defined as the smallest circle including 130,000 household addresses in the public directory. The size of these areas varied (5 to 5000 km^2^) according to population density (see Figure 1). In each area, two lists of households were drawn:

· A “landline” list of 25 households: these households were chosen as those with the phone number immediately following a landline number drawn in this area. Since landline numbers are geographically allocated, this method ensured reaching households who chose not to be listed in the national directory.

· A “mobile phone” list of 7 households: these households were directly drawn in the national directory, in order to reach households without a fixed phone.

Addresses were iteratively drawn from the 40 lists of 130,000 households each and these households were phoned to present the study and, upon meeting eligibility criteria, were sent a written description of the study. A household was considered as “pre-included” when a referent member sent back a filled form to confirm his agreement. According to this method, 1,280 households were pre-included, i.e. agreed to be visited by a nurse for an inclusion visit involving all household members. We anticipated that 20% of pre-included households would finally decline to participate in the cohort.

### Eligibility criteria

A household was defined as a person or group of people occupying the same domicile. All households were eligible to participate in the cohort, provided at least one member was over 18 years of age and French-speaking. A household member was defined as a person living at least half his/her time in the household. All household members were eligible, regardless of age. The inclusion of a household required the participation of all members: the refusal of one or more member(s) prevented the inclusion of other members.

### Participants

**Figure 2:**
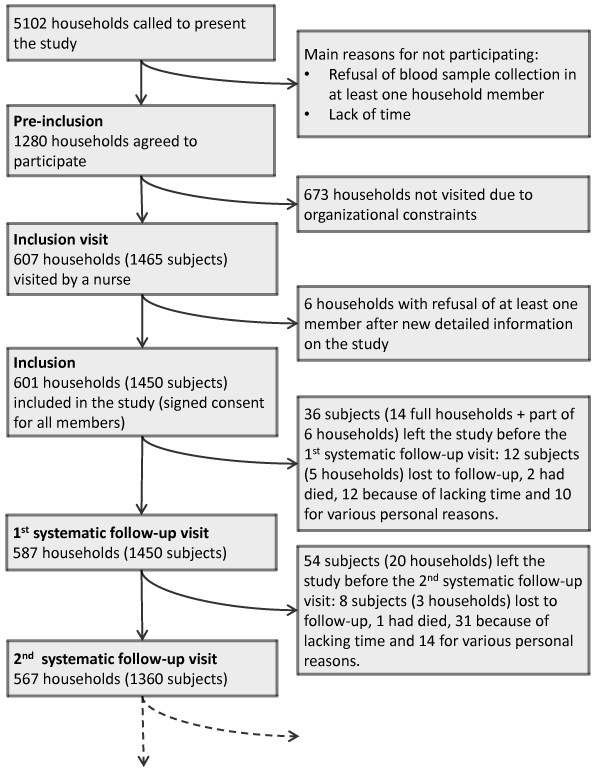
**Flow diagram until the 2**^**nd**^**systematic follow-up visit.**

Five thousand one hundred and two households were contacted by phone in order to achieve our targeted number of 1280 pre-included households (see Figure 2). The rate of contacted eligible households who agreed to be pre-included varied from 17% to 34% across the 40 areas. The main reasons for non-participation were lack of time and expected difficulty to collect blood samples from children.

Six hundred and seven households were visited by a nurse for an inclusion visit, among which six finally did not agree to participate (refusal of at least one member after receiving more detailed information on the study). Data was collected on the 601 remaining households (1450 subjects). According to population census data [20], these households had sociodemographic characteristics close to the general population (see Additional file 1: Figure S1 and Additional file 1: Tables S1–S6 for details).

### Data collection

**Figure 3:**
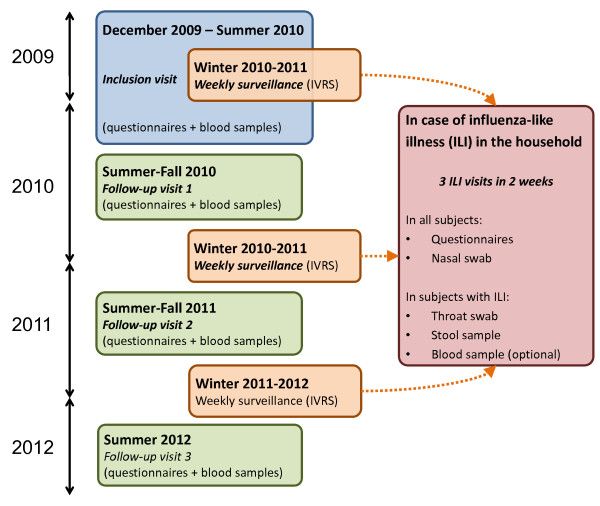
Design of the study: systematic and additional visits of households by nurses.

The main objective of this study was to identify individual and collective determinants of H1N1pdm infection; therefore we tried to collect comprehensive data about subjects and their environment, in addition to biological samples. Several household visits are carried on by nurses for this purpose (see Figure 3 for details).

**Figure 4:**
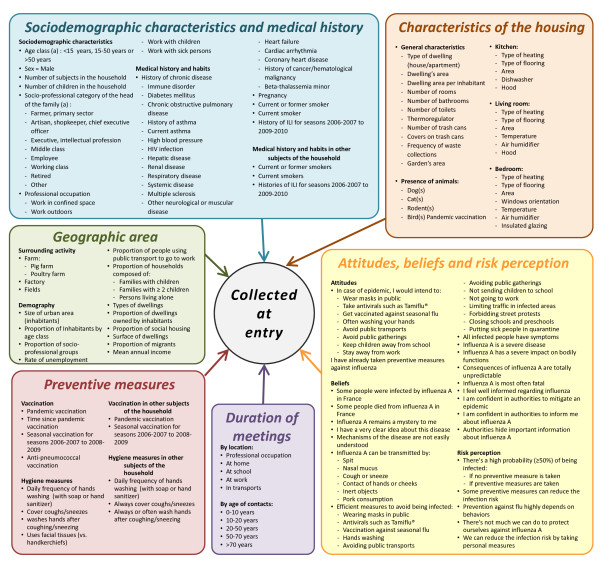
Main data collected on questionnaires at entry in the cohort, in addition to blood samples.

· Inclusion visits During the inclusion visit, nurses collected from all subjects detailed data regarding medical history, vaccination and preventive measures against influenza, smoking habits, socioeconomic status, risk perception and beliefs, frequency and characteristics of meetings with other people and housing (personal room, house or apartment). As the households’ addresses were geocoded, we were able to get additional information from public databases regarding the immediate surrounding environment of households. An overview of data collected from questionnaires at entry in the cohort is shown in Figure 4. Blood samples were collected and centralized for serological analyses. For subjects over 10 years, a heparinated tube was also collected to study cellular immunity, as well as a blood sample dedicated to transcript analyses.

· Systematic yearly visits After the inclusion visit, systematic follow-up visits are carried on between influenza seasons. During a systematic visit, a nurse collects or updates individual and environmental data on questionnaires, completes previously missing data, and obtains blood samples from all members of the household. Two waves of systematic follow-up visits have already occurred (summer-fall 2010 and 2011). A third wave is expected by the end of the second year of follow-up (summer 2012).

· Influenza-like illness (ILI) visits During the influenza season (as defined by the French surveillance network [21]), we use an active surveillance system order to detect ILIs: all households are called by an interactive voice response system (IVRS) weekly and are asked if any subject has symptoms of ILI (fever ≥ 37.8°C associated with cough or sore throat, as defined by the CDC [22]). A free phone number is given to subjects to report symptoms spontaneously between two weekly calls. In case of reported ILI, symptoms are validated by the study team and then three “ILI visits” are organized: nurses visit the household within 48 h after the onset of symptoms, then 3–6 days and 8–12 days after the onset.

· During these visits, a detailed questionnaire collects data about the circumstances of possible exposure to influenza viruses and the chronology of symptoms (if any) in all subjects. Nasal swabs are collected from all subjects. A stool sample and a throat swab are also collected from subjects with ILI, as well as a blood sample from those over 10 years of age. Moreover, a self-swab procedure is previously sent to the households in order to collect virological samples when a visit by a nurse within the first 48 h is not possible. Nasal swabs are used to identify various respiratory viruses by PCR and biochips allowing for multiple diagnosis tests.

· This series of three visits can occur several times in the same household during an influenza season. There were 23 ILI alerts during the 2009–2010 season (as households were still being included) and 143 during the 2010-2011 season, all of which triggered up to three ILI visits.

· Vaccination visits In order to update serological information, a blood sample was collected from subjects who had an influenza vaccination, between 2 and 4 weeks following this vaccination. There was one vaccination visit following the inclusion visits; 29 vaccination visits were conducted following the first wave of follow-up visits and 69 following the second wave.

### Timeline

The cohort was initially designed to include households before the 2009 pandemic season and to follow subjects during the two subsequent influenza seasons. We obtained funding in June 2009, the cohort protocol and questionnaires were finalized in July 2009, and the protocol obtained ethical approval on September 8, 2009. Households were pre-included between September 25 and December 17, 2009 and inclusion visits began on December 4, 2009 – as the final administrative authorizations were obtained. The inclusion period was extended until July 31, 2010, in order to get a relevant sample size for the planned analyses, some of which were postponed until the following season. A total of 575 households (96%) were included after the first pandemic season (September 7 to December 27, 2009 [23]).

### Ethical considerations

The protocol of the CoPanFlu-France study was approved by the research ethics committee “Comité de Protection des Personnes Ile-de-France 1” on September 8, 2009. Information was previously given by investigators to participants indirectly through written descriptions of the study and training of the nurses, and directly by e-mail and telephone for any question. Written informed consent was obtained for all subjects.

## Discussion

### Expected results

Many analyses have been recently completed or are currently being carried out. Based on inclusion data, we used a data-driven approach to identify factors associated with a high anti-H1N1pdm serological titer. We are conducting several analyses to identify risk factors associated with influenza infections (based on serological data for the first pandemic season, then on both serological and virological data for the following seasons). Nasal swabs are being analyzed to identify various respiratory viruses and the characteristics of infected subjects. Blood samples collected during ILI visits are used to study innate immunity against influenza and the related transcriptome. Determinants of vaccination against influenza have also been identified, and other studies are being conducted in the field of social science and risk perception. Several other analyses are expected soon from different collaboration partners in various biomedical fields.

### Strengths of the study

The main strength of this cohort is the large amount of available and expected data and the different biological samples to be collected, which will permit to carry on many studies in various biomedical fields. To our knowledge, this project is the first attempt to study so thoroughly the determinants of infections by respiratory viruses in a large sample of households randomly selected in the community. This approach is likely to provide new insights from the interaction of sparse data usually studied separately, especially with the help of data-driven methods such as those already under development in the field of non-communicable diseases [15,16]. Additional data is being collected and analyzed in this ongoing cohort, whose longitudinal analysis will permit integrative analyses of complex phenomena such as individual, collective and environmental risk factors of infection, routes of transmission, or determinants of the immune response to infection or vaccination.

### Limitations

We designed the CoPanFlu-France study in order to enable inference to the French general population, yet we cannot exclude a selection bias induced by the proportion of contacted households who refused to participate. However, a comparison between CoPanFlu subjects and population census data [20] suggests that this bias was controlled (see supplementary material part 2).

We wish we were able to set up this project a few months earlier, in order to include households before and to follow-up subjects during the 2009 pandemic season. Due to organizational impairments, the inclusion process was delayed and data regarding ILIs were collected retrospectively, sometimes up to 6 months after the epidemic. Thus, this timeline of inclusion may have induced recall or reporting biases for the 2009 season, and we were not able to collect enough pre-pandemic blood samples and nasal swabs during this first H1N1pdm season to investigate laboratory-confirmed infections. Another consequence of this delayed inclusion process is that we decided to stop inclusions as only 601 out of the 1000 expected households were included. This limit is the main reason why we decided to postpone the end of the study until 2012 instead of 2011 as initially expected, in order to collect data during an additional influenza season.

## Competing interests

Pr Carrat reported not having shares or paid employment with pharmaceutical companies; received honoraria from Novartis, GlaxoSmithKline and Boiron and received travel support to attend scientific meetings from Novartis. Other authors have no competing interest to declare.

## Authors’ contributions

NL, FC and AF drafted the protocol of the study. All authors contributed to conception and design of the study. NL, PF, MSe, RMD and FC participated to the acquisition of data. NL and FC carried out the statistical analyses. NL and FC drafted the manuscript and all authors revised it critically. All authors gave final approval of this version of the manuscript.

## Pre-publication history

The pre-publication history for this paper can be accessed here:

http://www.biomedcentral.com/1471-2458/12/417/prepub

## Supplementary Material

Additional file 1 Design and methods of the CoPanFlu-France cohort: representativeness of the population sample. Click here for file

## References

[B1] Dawood FS Jain S Finelli L Shaw MW Lindstrom S Garten RJ Gubareva LV Xu X Bridges CB Uyeki TM Emergence of a novel swine-origin influenza A (H1N1) virus in humans N Engl J Med 2009 360 2605 2615 19423869 10.1056/NEJMoa0903810

[B2] **World now at the start of 2009 influenza pandemic** [http://www.who.int/mediacentre/news/statements/2009/h1n1_pandemic_phase6_20090611/en/index.html].

[B3] Koita OA Sangare L Poudiougou B Aboubacar B Samake Y Coulibaly T Pronyk P Salez N Kieffer A Ninove L Flahault A de Lamballerie X A seroepidemiological study of pandemic A/H1N1(2009) influenza in a rural population of Mali Clinical Microbiology and Infection: The Official Publication of the European Society of Clinical Microbiology and Infectious Diseases 2011 10.1111/j.1469-0691.2011.03725.x 22221838

[B4] Dellagi K Rollot O Temmam S Salez N Guernier V Pascalis H Gérardin P Fianu A Lapidus N Naty N Tortosa P Boussaïd K Jaffar-Banjee M-C Filleul L Flahault A Carrat F Favier F de Lamballerie X Pandemic influenza due to pH1N1/2009 Virus: estimation of infection burden in Reunion Island through a prospective serosurvey, Austral Winter 2009 PLoS One 2011 6 e25738 10.1371/journal.pone.0025738 21980532 PMC3183080

[B5] Cauchemez S Donnelly CA Reed C Ghani AC Fraser C Kent CK Finelli L Ferguson NM Household transmission of 2009 pandemic influenza A (H1N1) virus in the United States N Engl J Med 2009 361 2619 2627 10.1056/NEJMoa0905498 20042753 PMC3840270

[B6] France AM Jackson M Schrag S Lynch M Zimmerman C Biggerstaff M Hadler J Household transmission of 2009 influenza A (H1N1) virus after a school-based outbreak in New York City, April-May 2009 J Infect Dis 2010 201 984 992 10.1086/651145 20187740

[B7] Lee DH Kim CW Kim J-H Lee JS Lee MK Choi JC Choi BW Choi S-H Chung J-W Risk factors for laboratory-confirmed household transmission of pandemic H1N1 2009 infection Am J Infect Control 2010 38 e43 e45 21093687 10.1016/j.ajic.2010.05.017

[B8] Morgan OW Parks S Shim T Blevins PA Lucas PM Sanchez R Walea N Loustalot F Duffy MR Shim MJ Guerra S Guerra F Mills G Verani J Alsip B Lindstrom S Shu B Emery S Cohen AL Menon M Fry AM Dawood F Fonseca VP Olsen SJ Household transmission of pandemic (H1N1) 2009, San Antonio, Texas, USA, April-May 2009 Emerging Infect Dis 2010 16 631 637 10.3201/eid1604.091658 20350377 PMC3321969

[B9] Nishiura H Oshitani H Household Transmission of Influenza (H1N1-2009) in Japan: age-specificity and reduction of household transmission risk by Zanamivir Treatment J Int Med Res 2011 39 619 628 21672367 10.1177/147323001103900231

[B10] Viboud C Boëlle P-Y Cauchemez S Lavenu A Valleron A-J Flahault A Carrat F Risk factors of influenza transmission in households Br J Gen Pract 2004 54 684 689 15353055 PMC1326070

[B11] Cowling BJ Chan K-H Fang VJ Cheng CKY Fung ROP Wai W Sin J Seto WH Yung R Chu DWS Chiu BCF Lee PWY Chiu MC Lee HC Uyeki TM Houck PM Peiris JSM Leung GM Facemasks and hand hygiene to prevent influenza transmission in households: a cluster randomized trial Ann Intern Med 2009 151 437 446 19652172 10.7326/0003-4819-151-7-200910060-00142

[B12] Simmerman JM Suntarattiwong P Levy J Jarman RG Kaewchana S Gibbons RV Cowling BJ Sanasuttipun W Maloney SA Uyeki TM Kamimoto L Chotipitayasunondh T Findings from a household randomized controlled trial of hand washing and face masks to reduce influenza transmission in Bangkok, Thailand Influenza Other Respi Viruses 2011 5 256 267 10.1111/j.1750-2659.2011.00205.x 21651736 PMC4634545

[B13] Brankston G Gitterman L Hirji Z Lemieux C Gardam M Transmission of influenza A in human beings Lancet Infect Dis 2007 7 257 265 10.1016/S1473-3099(07)70029-4 17376383

[B14] Coudeville L Bailleux F Riche B Megas F Andre P Ecochard R Relationship between haemagglutination-inhibiting antibody titres and clinical protection against influenza: development and application of a bayesian random-effects model BMC Med Res Methodol 2010 10 18 10.1186/1471-2288-10-18 20210985 PMC2851702

[B15] Bougnères P Valleron A-J Causes of early-onset type 1 diabetes: toward data-driven environmental approaches J Exp Med 2008 205 2953 2957 10.1084/jem.20082622 19075294 PMC2605242

[B16] Patel CJ Bhattacharya J Butte AJ An Environment-Wide Association Study (EWAS) on type 2 diabetes mellitus PLoS One 2010 5 e10746 10.1371/journal.pone.0010746 20505766 PMC2873978

[B17] Waksberg J Sampling Methods for Random Digit Dialing J Am Stat Assoc 1978 73 40 46 10.1080/01621459.1978.10479995

[B18] Dudoignon L Vanheuverzwyn A Coverage Optimization of the Telephone Surveys Thanks to the Inclusion of Mobile Phone Only Stratum

[B19] Roy G Vanheuverzwyn A Mobile phone in sample surveys 2002 Proceedings from International Conference on Improving Surveys (ICIS), Copenhagen (Denmark) 25–28 August

[B20] **Databases - The population census** [http://www.insee.fr/en/bases-de-donnees/default.asp?page=recensements.htm].

[B21] **Sentinelles network France - Weekly epidemiological record** [http://sentiweb.org/].

[B22] **Case Definitions for Infectious Conditions Under Public Health Surveillance** [http://www.cdc.gov/osels/ph_surveillance/nndss/casedef/novel_influenzaA.htm].

[B23] Carrat F Pelat C Levy-Bruhl D Bonmarin I Lapidus N Planning for the next influenza H1N1 season: a modelling study BMC Infect Dis 2010 10 301 10.1186/1471-2334-10-301 20964814 PMC2975658

