# Supplementary material for: Integrative study of pandemic A/H1N1 influenza infections: design and methods of the CoPanFlu-France cohort
Source: BMC Public Health. 2012 Jun 7;12:417. doi: 10.1186/1471-2458-12-417 (PMC3461458; doi:10.1186/1471-2458-12-417)
Supplement: Additional file 1 — Design and methods of the CoPanFlu-France cohort: representativeness of the population sample. [file 1471-2458-12-417-S1.doc]

**Additional material**

**Integrative study of pandemic A/H1N1 influenza infections: design and methods of the CoPanFlu-France cohort**

Nathanael Lapidus, Xavier de Lamballerie, Nicolas Salez, Michel Setbon, Pascal Ferrari, Rosemary M. Delabre, Marie-Lise Gougeon, Frédéric Vely, Marianne Leruez-Ville, Laurent Andreoletti, Simon Cauchemez, Pierre-Yves Boëlle, Eric Vivier, Laurent Abel, Michaël Schwarzinger, Michèle Legeas, Pierre Le Cann, Antoine Flahault, Fabrice Carrat

**Table of contents:**

1. **Representativeness of the population sample 2**

Supplemental table 1: Age 2

Supplemental table 2: Sex 2

Supplemental figure 1: Age structure diagram 2

Supplemental table 3: Size of households 3

Supplemental table 4: Size of urban areas 3

Supplemental table 5: Socio-professional group of the head of the family 3

Supplemental table 6: Pandemic vaccine coverage 4

1. **Calculation of sampling weights 5**
2. **Representativeness of the population sample**

The subjects included in the CoPanFlu-France study were compared to the French general population (2009 census by French Institut national de la statistique et des études économiques – Insee(1)), at either the household or the individual level.

Supplemental table 1: Age of CoPanFlu subjects in comparison to French general population (1)

| **Age** | **CoPanFlu subjects** | **Insee 2009 (×10^3^ )** |
| --- | --- | --- |
| **0-10 years** | 184 (12.7%) | 7805 (12.3%) |
| **10-20 years** | 194 (13.4%) | 7915 (14.4%) |
| **20-30 years** | 118 (8.1%) | 7960 (12.5%) |
| **30-40 years** | 179 (12.3%) | 8667 (13.6%) |
| **40-50 years** | 217 (15.0%) | 8930 (14.0%) |
| **50-60 years** | 199 (13.7%) | 8537 (13.4%) |
| **60-70 years** | 216 (14.9%) | 5805 (9.1%) |
| **70-80 years** | 116 (8.0%) | 4867 (7.7%) |
| **80-90 years** | 27 (1.9%) | 2636 (4.1%) |
| **> 90 years** | 0 (0.0 | 480 (0.8%) |
| ***Total*** | *1450 (100%)* | *63602 (100%)* |

Supplemental table 2: Sex of CoPanFlu subjects in comparison to French general population (1)

| **Sex** | **CoPanFlu subjects** | **Insee 2009 (×10^3^ )** |
| --- | --- | --- |
| **Males** | 685 (47.2%) | 30797 (48.4%) |
| **Females** | 765 (52.8%) | 32805 (51.6%) |
| ***Total*** | *1450 (100%)* | *61795 (100%)* |

Supplemental figure 1: Age structure diagram of CoPanFlu subjects (histograms) and expected number of subjects with respect to French general population (curves) (1)
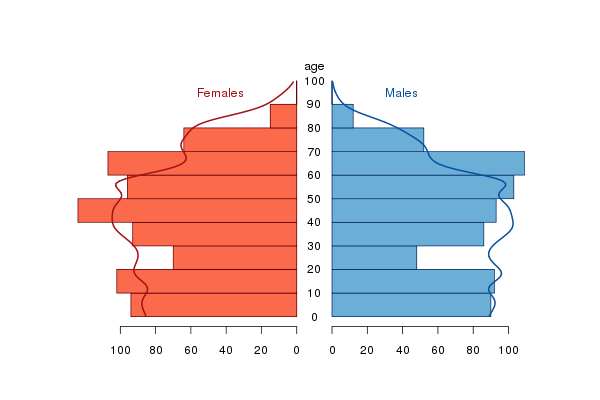


Supplemental table 3: size of CoPanFlu households in comparison to French general population (1)

| **Size of households** | **CoPanFlu subjects** | **Insee 2009 (×10^3^ )** |
| --- | --- | --- |
| **1** | 142 (23.6%) | 8765 (33.3%) |
| **2** | 250 (41.6%) | 8667 (32.9%) |
| **3** | 85 (14.1%) | 3900 (14.8%) |
| **4** | 83 (13.8%) | 3300 (12.5%) |
| **≥ 5** | 41 (6.8%) | 1721 (6.5%) |
| ***Total*** | *601 (100%)* | *26352 (100%)* |

Supplemental table 4: size of urban areas for CoPanFlu households in comparison to French general population (1)

| **Type of rural/urban area** | **CoPanFlu subjects** | **Insee 2009 (×10^3^ )** |
| --- | --- | --- |
| **Rural** | 325 (22.4%) | 15806 (24.9%) |
| **Urban area < 5000 inhabitants** | 74 (5.1%) | 3485 (5.5%) |
| **5000 < urban area < 20000 inhab** | 138 (9.5%) | 7108 (11.2%) |
| **20000 < urban area < 100000 inhab** | 246 (17.0%) | 8809 (13.8%) |
| **urban area > 100000 inhab (except Paris metropolitan area)** | 454 (31.3%) | 18196 (28.6%) |
| **Paris metropolitan area** | 213 (14.7%) | 10198 (16.0%) |
| ***Total*** | *1450 (100%)* | *63602 (100%)* |

Supplemental table 5: socio-professional group of the head of the family for CoPanFlu households in comparison to French general population (1)

| **Socio-professional group** | **CoPanFlu subjects** | **Insee 2009 (×10^3^ )** |
| --- | --- | --- |
| **Farmer, primary sector** | 15 (1.0%) | 1065 (1.7%) |
| **Artisan, shopkeeper, chief executive officer** | 45 (3.1%) | 3622 (5.8%) |
| **Executive, intellectual profession** | 234 (16.1%) | 7980 (12.8%) |
| **Middle class** | 282 (19.4%) | 9948 (16.0%) |
| **Employee** | 212 (14.6%) | 7331 (11.8%) |
| **Working class** | 227 (15.7%) | 13774 (22.2%) |
| **Retired** | 376 (25.9%) | 14902 (24.0%) |
| **Other** | 59 (4.1%) | 3493 (5.6%) |
| ***Total*** | *1450 (100%)* | *62116 (100%)* |

Supplemental table 6: pandemic vaccine coverage for CoPanFlu households in comparison to French general population (2)

| **Age** | **CoPanFlu subjects** | **General population** |
| --- | --- | --- |
| **0-10 years** | 29/184 (15.8%) | 15.7% |
| **10-20 years** | 24/194 (12.4%) | 8.9% |
| **20-30 years** | 5/118 (4.2%) | 5.1% |
| **30-40 years** | 23/179 (12.8%) | 8.7% |
| **40-50 years** | 20/217 (9.2%) | 6.3% |
| **50-60 years** | 25/199 (12.6%) | 6.3% |
| **60-70 years** | 26/216 (12.0%) | 8.7% |
| **70-80 years** | 21/116 (18.1%) | 7.0% |
| **> 80 years** | 3/27 (11.1%) | 6.0% |
| ***Total*** | *176/1450 (100%)* | *100%* |

1. **Calculation of sampling weights**

For all the 40 areas inside which subjects were eligible, we estimated the number of people in the general population stratified by sex, age, size of the household, socio-professional group and size of the urban area (1). For all these strata, we estimated the number of people owning a fixed or mobile phone, thanks to data published by the French research center for study and observation of living conditions (Crédoc) (3). We then estimated the probabilities of reaching a given household in each of these strata, by either fixed or mobile phone (with regard to the estimated number of mobile phones per household) by dialing 25 fixed phone numbers and 7 mobile phone numbers respectively. These probabilities were our proxies for probabilities of inclusion, whose inverse set initial sampling weights

We retrieved from the 2009 census (1) and from other published data (2) the distribution of the French general population according to age, sex, size of the household, size of the urban area, socio-professional group of the head of the family and pandemic vaccine coverage by age group (see supplemental tables 7-12). These auxiliary covariates were used to reweigh the survey design with the help of the function “calibrate” from the R package “survey,” (4) relying on a generalized raking procedure (5). These weights were those used for the calculation of post-stratified estimates.

**References:**

1. Insee. Databases - The population census [Internet]. 2011 [cité 2011 janv 19];Available from: http://www.insee.fr/en/bases-de-donnees/default.asp?page=recensements.htm

2. Carrat F, Pelat C, Levy-Bruhl D, Bonmarin I, Lapidus N. Planning for the next influenza H1N1 season: a modelling study. BMC Infect Dis. 2010 oct 21;10(1):301.

3. La diffusion des technologies de l’information et de la communication dans la société française (2009) - La Documentation française [Internet]. [cited 2010 oct 18];Available from: http://www.ladocumentationfrancaise.fr/rapports-publics/094000589/index.shtml

4. Lumley T. Complex Surveys: A Guide to Analysis Using R. John Wiley and Sons; 2010. 296 p.

5. Deville J-C, Sarndal C-E, Sautory O. Generalized Raking Procedures in Survey Sampling. Journal of the American Statistical Association. 1993 sept;88(423):1013–20.
